# Decoding the chaffinch “rain” call: a female-directed alarm call?

**DOI:** 10.1093/beheco/araf039

**Published:** 2025-05-04

**Authors:** Léna de Framond, Rebecca Müller, Alberto Comin, Henrik Brumm

**Affiliations:** Animal Communication & Urban Ecology Group, Max Planck Institute for Biological Intelligence, Eberhard-Gwinner-Straße, 82319 Seewiesen, Germany; Animal Communication & Urban Ecology Group, Max Planck Institute for Biological Intelligence, Eberhard-Gwinner-Straße, 82319 Seewiesen, Germany; Animal Communication & Urban Ecology Group, Max Planck Institute for Biological Intelligence, Eberhard-Gwinner-Straße, 82319 Seewiesen, Germany; Animal Communication & Urban Ecology Group, Max Planck Institute for Biological Intelligence, Eberhard-Gwinner-Straße, 82319 Seewiesen, Germany

**Keywords:** animal communication, bird call, bird song, *Fringilla coelebs*, rain call

## Abstract

Acoustic communication is vital for many animal taxa. Many songbirds have elaborate communication systems and large vocal repertoires consisting of learned, complex songs, and calls that are usually simpler in structure. While the functions of songs have been well researched, the functions of bird calls are often difficult to deduce from the context. A well-known example is the “rain” call of the common chaffinch (*Fringilla coelebs*): although chaffinches are very common and “rain” calls are conspicuous and frequent, the function of this call is still a mystery. It has been proposed to serve such diverse functions such as song substitute in territorial contests, predator alarm call, or within-pair coordination. Here, we systematically tested these hypothesized three functions, using a combination of two playback experiments and field observations. We found that chaffinches did not react to “rain” call playbacks with the same aggressive behavior as to song playbacks. Predator vocalizations, however, consistently elicited “rain” calls. In addition, when a female was visible, male chaffinches used “rain” calls more often both during predator simulation and in the actual presence of predators. Since the “rain” call is only uttered during the breeding season and it is associated with the presence of a female and predators, we propose that the “rain” call is a specific alarm call used in the context of defense against nest predators.

## Introduction

Birds, and passerines in particular, use a variety of acoustic signals to coordinate and facilitate crucial tasks such as mate attraction and mate choice, resource acquisition and defense, predation avoidance, and social bonding. Traditionally, songbird vocalizations were divided into songs, which are learned complex signals used to attract mates and deter rivals, and calls, which are simpler, innate, and context-specific utterances (Catchpole and Slater 2008). For example, birds use alarm and mobbing calls to avoid threats by alerting nearby con- and hetero-specifics to the presence of predators (Templeton et al. 2005; Magrath et al. 2007; Randler and Förschler 2011). In gregarious species, some calls can also facilitate coordination within flocks during movement (Arnold et al. 2022) or foraging (Radford and Ridley 2008). Parent-offspring communication is another vital role, as illustrated by the begging calls of nestling birds (Wright and Leonard 2007) or the calls used to identify parents and offspring in a breeding colony (Aubin and Jouventin 2002). However, the function of many bird calls is still unclear. Recent work has even challenged the binary division of songbird vocalizations into songs and calls, highlighting a continuum of complexity, diversity of function, and division of sex roles in vocal behavior (Rose et al. 2021), and emphasizing the role of learning in call development (Elie and Theunissen 2020). A striking example of a well-known vocalization that does not fit into either the “call” or “song” category, and the function of which has not been clarified, is the “rain” call of the common chaffinch *Fringilla coelebs*.

**Fig. 1.**
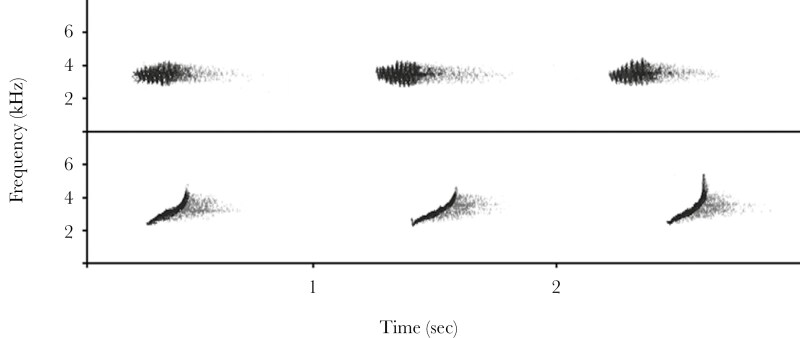
Spectrograms for the two “rain” call dialects most common in the study area: "rülsch" (upper panel) and "huit" (lower panel).

Chaffinches are among the most common European songbirds and the singing behavior of this species has been intensively studied (e.g., Hinde 1958; Slater 1981; Riebel and Slater 1999; Brumm et al. 2009; Lachlan et al. 2013; Yelimlieş et al. 2023). Their song is a loud, conspicuous series of two to four trills ending in a flourish (Slater and Ince 1979), uttered throughout the day in spring and summer (Bergmann 1993). Their equally conspicuous “rain” call consists of a single element (Fig. 1) that is typically repeated at a high rate (once every 1 to 3 s) for extended periods, sometimes up to 20 min (personal observation). The chaffinch “rain” call has caught the attention of researchers for a long time, not only because of its conspicuousness but also because it is unusual in terms of seasonality, usage, and ontogeny. Although the “rain” call has a typical call-like short and simple structure, it is only produced by males from inside the territory during the breeding season (Heyder 1954; Poulsen 1958; Bergmann 1993). Just as with song, male chaffinches also start to produce “rain” calls in spring, probably triggered by increased testosterone levels (Poulsen 1951). Moreover, and most unusual for a call, it is subject to vocal production learning, as suggested by the existence of various “rain” call dialects (Riebel and Slater 1998, see Fig. 1 for two examples). The geographic variation of “rain” call dialects has been recognized for over 300 yr (von Pernau 1707), and the small-scale, often mosaic-like distribution has been investigated in detail across Europe, eg in Denmark (Poulsen 1958), Finland (von Haartman and von Numers 1992), Germany (Sick 1939; Baptista 1990; Skiba 2005), Russia (Ivanitskii et al. 2021), and Ukraine (Tsvelykh and Yablonovska-Grishchenko 2013).

Despite this extensive body of research, the function of the “rain” call is still unclear. Three non-mutually exclusive hypotheses have been put forward: (1) The song-like properties of the “rain” call in conjunction with the name of the call, likely led to the song-substitute hypothesis. Chaffinches might use “rain” calls instead of songs in unfavorable conditions (Poulsen 1958; Skiba 2005; Budka et al. 2019) although there is no link to rain (Skiba 2005; de Framond et al. 2024). This entails that the “rain” call doubles at least some of the song functions, including territorial defense, as suggested by the increased “rain” call activity in response to song playback (Budka et al. 2019; Deoniziak and Osiejuk 2020). (2) An alternative hypothesis is related to a function as alarm call. Indeed “rain” calls are also observed in the presence of predators (Sick 1939; Peitzmeier 1955; Marler 1956a; Skiba 2005; Bērziņš et al. 2010) and they may even be used to grade mobbing response strength (Krams and Krama 2002). Moreover, anecdotal evidence that nestlings falling silent in response to “rain” calls further supports the notion of an alarm call (Marler 1956a; Davies et al. 2004). (3) Finally, “rain” calls are frequently heard when female chaffinches are seen in the direct vicinity of the focal males (Marler 1956b; de Framond et al. 2024) which would suggest that the “rain” call serves a function within the pair. Here, we combined two playback experiments with one observational study to test the three hypotheses about the function of the chaffinch “rain” call. To test the song-substitute / territorial defense hypothesis, we simulated territorial intrusions with song playbacks and with “rain” call playbacks. If “rain” calls is used similarly to songs in territorial interactions, we predicted that male chaffinches would respond to singing and “rain”-calling intruders with a similar, aggressive behavior. To test the alarm-call hypothesis, we observed the reaction of chaffinches to predator presence. If the “rain” call is indeed an alarm call, we predicted that the presence of predators or predator cues would elicit chaffinch “rain” calls. We also quantified the “rain” call probability in relation to the presence of a female in the direct vicinity of the focal bird to further investigate the social context for “rain” call usage. If the “rain” call is used for within-pair coordination, we predicted that males would increase the frequency of “rain” calls when their mate is visible.

## Methods

We investigated the function of the chaffinch “rain” call using two playback experiments and extensive field observations in the spring of 2023 and 2024 in parks and mixed forests in Bavaria, southern Germany (47°58’ N, 11°19’ E). In the first playback experiment, we observed the response of chaffinches to “rain” calls. In the second experiment, we measured “rain” call probability in response to predator presence. The most common “rain” call dialects in our study sites were the “huit” and “rülsch” type (Fig. 1).

### Playback 1: response to “rain” calls

We compared the responses of 15 chaffinch males to each of three types of playbacks: conspecific song, “rain” call (“rülsch” dialect, which was most common in 2022), and control (blackbird song). Before the experiments, we recorded wild chaffinches and blackbirds (*Turdus merula*) with a directional microphone (Sennheiser ME 66 with a RØDE Blimp MkII windshield), and Marantz Professional PMD660 solid-state recorder (sampling rate 44.1 kHz). These recordings were made at our field site and surrounding areas, but we made sure to not broadcast vocalizations of birds recorded within ~1 km of the tested males. Using the software Audacity (version 3.3), we obtained a 1-min-long snippet from each recording, or repeated a shorter snippet to reach 1 min, excluding loud noises, other birds’ songs and songs or “rain” calls from other chaffinches in the background. Recordings were high-pass filtered (2 kHz for “rain” calls and at 1.5 kHz for chaffinch and blackbird songs), the amplitude of each vocalization was adjusted to a peak amplitude of −1 dB full-scale. We added a 500 ms tone (0.5, 0.75 and 1 kHz) at the start, end, and 3 min after the end of each playback to help observers keep track of the start and end of the observation period during trials. Since song amplitude is an aggressive signal (Brumm and Ritschard 2011), we calibrated every recording using a Casella 240 V035-07 sound pressure level meter to reach a natural amplitude (mean ± SD (range) in dB(A) SPL re 20 µPa @ 1 m, measured as advised in Brumm and Zollinger (2011), natural range from unpublished data: blackbird: 88 ± 1.3 (86 to 91), natural range = 69 to 97; chaffinch song: 85 ± 2.2 (80 to 88), natural range = 77 to 89; “rain” call: 81 ± 3.7 (77 to 86), natural range = 74 to 89). In total, we prepared ten different playback renditions from ten different source males for each type.

The playback experiments took place from April, 18^th^ to May, 9^th^ 2023. Birds were sampled three times within 1 wk and no less than 2 h apart (median = 2.8 h, quartile = 2.3 - 15.8, range = 2.1 to 75.2, one exception for one bird which was tested in 13 d). Each trial consisted of three phases: 3 min of pre-playback observation, 1 min of playback, and 3 min of post-playback observation. We recorded four behavioral variables, which we spoke into a small audio recorder: “rain” calls, aggressive “chink” calls, complete songs (with a flourish) and incomplete songs. We started a trial upon localizing a consistently singing or “rain” calling chaffinch, to make sure we did not fall right in the fertile period of the female, where the male vocal behavior is very different (Hanski and Laurila 1993). Playbacks were presented from a Leike DJ Roxxx Round Clip XXPro speaker on a tripod, mounted one meter above the ground, placed as close to the bird as possible (7.1 to 27 m, mean = 16.4 m) and no longer than 5 min after the end of the pre-playback observation phase. We recorded the initial and minimum perched distance to the speaker with a laser range finder (Bresser 6 × 24 rangefinder & speedmeter). After each trial, we noted whether we saw a female nearby and heard a countersinging male, we measured the maximum ambient noise level amplitude over 15 s and the playback amplitude (dB(A), SPL meter Casella Cel-240 V035-07, fast mode). We calculated the received level of the playback at the bird’s initial position using the inverse square law (Dooling and Blumenrath 2013). Trials were considered successful when the bird was less than 25 meters away from the loudspeaker and/or the received level of the playback at the position of the bird was 10 dB above ambient noise. If these conditions were met, we assumed that the playback was most likely heard by the birds. We were able to match the “rain” call dialect of the focal bird with that of the playback in only in 4 cases out of 15 because most birds in 2023 used the “huit” dialect, contrarily to the prevailing “rülsch” type in the previous year when we recorded the playback stimuli. The difference between responses of birds with matching and non-matching dialect is shown in Fig. S2. From the audio recordings, we counted flights, “rain” calls, chinks, and complete and incomplete songs per playback phase (ie before, during, and after playback).

### Playback 2: response to predator vocalizations

To investigate a potential role of the “rain” call as an alarm call, we measured the reaction of 20 wild chaffinches to three types of playbacks simulating the presence of two predators (spectrograms in Fig. S1): calls of the Eurasian sparrow hawk (*Accipiter nisus*), which is a danger to adult chaffinches and occasionally preys on nestlings (Newton 2010), and the carrion crow (*Corvus corone*), which is a danger for eggs and nestlings as corvids are responsible for about 10 % of all nest predation events (DeGregorio et al. 2016). We used the song of the common blackbird, a harmless species, as a control. We exposed chaffinches to 5 min of playback with increasing intensity, simulating approaching danger, and measured the time it took them to initiate “rain” call behavior, thereby testing both if “rain” calls are consistently triggered by predator presence and if “rain” call intensity scales with danger level. While we did not subject the birds to a realistic predation situation (it is unlikely that a sparrowhawk would call continuously for 5 min from the ground), we capitalized on the tendency of songbirds to react strongly to any kind of predation-like situation (e.g. even to low level threats such as sleeping owls during the day).

Experiments took place between May, 14^th^ and May, 29^th^ 2024. Birds were tested three times each, 2 h apart and within 3 d (median = 2.5 h, quartile = 2.1 to 3.2, range = 1.75 to 21.9). We obtained five high-quality crow recordings and four high-quality hawk recordings from the online database Xeno-Canto, from which we extracted audio which we high-pass filtered (500 Hz for hawks, no filter for crows) and looped when necessary to obtain a 1-min snippet. We normalized each vocalization to the same amplitude, then repeated the snippet four additional times and increased the amplitude by 3 dB every minute, which resulted in a 5-min long file with amplitudes ranging from −16 dB (0 to 60 s) to −1 dB (240 to 300 s) full scale. Playbacks were calibrated so that the final playback source level ranged from 80 to 95 dB (A) SPL re 20 µPa @ 1 m across the 5 min. We started trials upon finding a continuously singing chaffinch (at least three songs in a row, no “rain” calls). Trials began with a 1-min period during which the natural number of songs and flights were recorded on a small audio recorder, and subsequently used as a baseline in the analysis. We then broadcasted the playback from a loudspeaker (JBL charge 5) placed on the ground as close to the bird as possible (4.1 to 26.6 m, mean = 11.47 m). During this phase, we recorded the number of flights and songs. If a “rain” call was heard, the playback was immediately halted to measure the birds’ response intensity, and the trial proceeded to a final 1-min observation period to record the number of flights, songs, and “rain” calls. If no “rain” call was heard throughout the playback, we kept describing the bird’s behavior for an additional minute. For each trial, we documented environmental parameters including ambient noise, the initial distance between the loudspeaker and the bird, and whether we saw a female within 5 m of the focal male.

### Behavioral observations

To figure out in which natural context chaffinches use “rain” calls, we closely observed five male chaffinches and mapped their “rain” call and song posts between May, 12^th^ and June, 7^th^ 2023. We observed each bird for eight sessions of four ten-min blocks each. We started a 10-min observation block upon localizing a vocalizing chaffinch and documented the number of “rain” calls, songs, and aggressive “chink” calls per 50-s bins (with a 10-s break to write down), and reported the last position at which the bird was vocalizing on a map. We also noted if another male was singing, and if a female or predators were nearby. We noted the presence of predators (ie crow, sparrowhawk, goshawk) when we saw one or when we heard alarm calls from other species within the space used by the focal bird. We waited for at least 5 min or until the bird changed perch or activity before starting a new block to decrease temporal correlation among blocks.

### Statistical analyses

All statistical analyses were performed in a Bayesian framework, with R version 4.2.2 using the package *rstanarm* version 2.32.1. We fitted ten Generalized Linear Mixed Models (GLMM) in total. All models were run with four cores and four chains, using the package’s default, weakly informative priors (Gabry and Goodrich 2020) and with the seed 12345 to ensure reproducibility. Information on model parametrization (model family and link function, number of iterations, warmup iterations, thin rate) is summarized in Table 1. Because the structure of some of our behavioral responses resembles that of survival data in medical studies (simultaneous measure of the occurrence probability of an event—the bird emitted “rain” calls—and the latency until it occurred), we applied Time-to-Event models (*stan-surv* from the *rstanarm* package) following (Brilleman 2019) to these responses. We compared Time-to-Event models with different baseline hazard distributions using Leave-One-Out (LOO) comparison and present the model with the lowest Expected Log-Predicted Density (ELPD). For all models, we ensured that chains had mixed correctly, that the model family was appropriate and gave sensible results by inspecting carefully the MCMC trace and posterior predictive plots, and checked model stability and sensitivity to specific observations using Leave-One-Out cross-validation with Pareto smoothed importance sampling (PSIS-LOO) (package *bayesplot* version 4.2.2) following guidelines from (Gabry 2022, 2024; Gabry and Modrák 2024a, 2024b). For each model, we repost the mean of marginal posterior distributions as an estimate of the effect size, and the 0.025% and 0.975% as a measure of the credible interval.

**Table 1.** Model parameters of the playback analyses.

Model/Response variable		Family	Link function	Iteration (warmup)	Thin rate
**Response to “rain” call playback**					
M1	Number of full songs	Negative binomial	Inverse	5000 (1000)	4
M2	Presence of “rain” calls after playback	Binomial	Logit	5000 (1000)	4
M3	Number of “chink” aggressive calls after playback start	Negative binomial	Inverse	5000 (1000)	2
M4	Approach probability/latency	Survival model with Weibull hazard baseline	-	5000 (1000)	10
M5	Minimum distance	Gaussian	Identity	5000 (1000)	4
**Response to predator playback**					
M6	Presence of “rain” calls	Binomial	Logit	2000 (1000)	3
M7	Latency to “rain” call (proportion of the intended playback time)	Binomial	Logit	2000 (1000)	3
M8	Number of “rain” calls	Poisson	Log	4000 (1000)	4
M9	Time-to-”rain” call	Survival model with Weibull hazard baseline	-	2000 (1000)	10
**Behavioral observation**					
M10	Presence of rain calls	Binomial	Logit	4000 (1000)	4

We analyzed the response of chaffinches to “rain” call playbacks (Playback 1) using five independent Generalized Linear Mixed Models (M1 to M5), one for each behavioral response. In M1, we modeled the number of complete songs as a function of the playback type (control, “rain” call, and song), playback phase (before, during, and after broadcast), and their interaction, using a negative binomial GLMM. Because the sample size of tested birds was too low, we could not test whether the birds reacted differently depending on whether the “rain” call dialect matched their own dialect. In M2, we modeled “rain” call presence after playback start as a binomial variable according to playback type, and whether the bird was already “rain” calling before we started the playback. In M3, we modeled the number of “chink” alarm calls as a function of playback type, phase, and their interaction with a negative binomial GLMM. In M4, we modeled the probability that the bird approached the loudspeaker according to playback type using a time-to-event model with a Weibull Hazzard baseline. Finally, in M5 we modeled the minimum distance from the bird to the loudspeaker according to the playback type with a Gaussian GLMM. In all models, we included whether the female was visible (0/1) as a fixed effect, since this parameter may influence “rain” call behavior (de Framond et al. 2024). To control for differences in detection probability and perceived signal intensity, we included the initial distance (centered on the mean = 15 m) and playback amplitude (centered on the mean and scaled to the standard deviation per playback type) as fixed effects. We further added the time to set up the loudspeaker as a fixed effect to account for natural behavioral changes across time: Since we started a trial only when the focal bird was consistently vocalizing, we expected the number of vocalizations to decrease if the bird changed behavior. To account for repeated measures from the same bird, we included the bird identity as a random effect. To compare the effects of the different playbacks among each other, we calculated contrasts in post-hoc tests using the *hypothesis()* method in the package *brms* (version 2.19.0) following (Vuorre 2020).

To investigate the response of chaffinches to predator presence (Playback 2), we used two different approaches. First, we modeled the “rain” call probability during the playback (0/1, M6), the latency to “rain” call (as a proportion of the total playback duration, M7), and the number of “rain” calls after playback stopped (normalized by the remaining time of the experiment; if birds started using “rain” calls only during the last minute, the time left for counting “rain” calls was shorter, M8) in the subsample of data in which the birds did “rain” call. We included playback type (control, crow, and hawk), whether the female was visible, ambient noise (centered on the mean and divided by 6—as 6 dB is equivalent to doubling the acoustic energy), the initial distance between the bird and the playback source, as fixed effects and the bird identity as a random effect to account for individual variability. We additionally added the flight activity (number of flights per minute during the trial) in the model investigating “rain” call probability because birds that flew a lot were often foraging and probably in a different behavioral state. Mobbing might also comprise increased flying activity, but our experiment never elicited any mobbing behavior in the focal birds. In particular, during the trials we did not observe any other behavior than foraging that was associated with high flight activity. We also added the time left for “rain” calling (accounting for the limitations imposed by the duration of our trial) as fixed effect in the model investigating “rain” call number. Second, we applied a Time-to-Event model (M9) to measure the probability that birds “rain” called across time in relation to the playback type, whether we saw the female, the initial distance, and ambient noise as a fixed effect, and the bird identity as a random factor.

Last, we examined the context in which “rain” calls occurred (behavioral observations). To reduce temporal autocorrelation, we summarized the occurrence of “rain” calls per 10-min observation block. We modeled the occurrence of “rain” call as a function of female detection during the 10-min block (number of minutes with female seen), other males’ song (number of minutes when song heard), and predator presence (number of minutes with predator) in a binomial GLMM (M10). We accounted for daily variation in bird activity by adding the time of day as a fixed effect, and for individual variation among birds by including territory ID and session as a random effect.

## Results

**Fig. 2.**
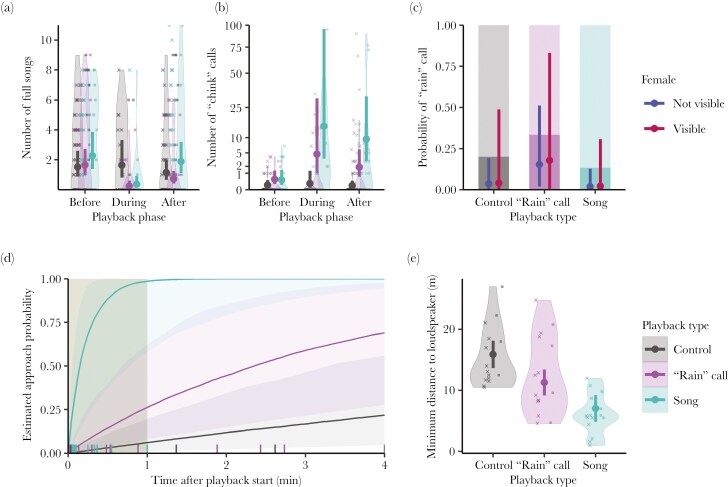
Chaffinches react to “rain” call playbacks less aggressively than to song playbacks. a) Number of songs and b) number of “chink” calls in each playback phase (before, during, and after broadcast), playback signal is color-coded (black: control, lilac: “rain” call, green: song). (c) Probability of hearing “rain” calls during and after the broadcast according to playback type and female detection by observers during the trial (point color). (d) Estimated (posterior mean and 95% credible interval) probability of approach across time. (e) Minimum distance to the loudspeaker during the playback as a function of the playback type. a, b, c, e) Large dots and error bars represent to model posterior means and 95% credible intervals. a, b, e) Raw data and data distribution are depicted as squares (“rain” call dialect matching playback) and crosses (“rain” call dialect not matching playback), and violin plots in the background. c) The proportion of playback trials with “rain” calls (dark bars) and without “rain” calls (light bars) are plotted in the background. d) Raw data depicted with the short vertical bars at the x-axis. The playback broadcast period is grayed out.

In Playback 1, we tested the reaction of 15 wild European chaffinches to “rain” call playbacks, compared to conspecific songs. Playing back chaffinch vocalization elicited a strong response from the birds in that they almost stopped singing (Table 2, M1, ß _Playback song: phase after_ = 1.94 (−3.13, −0.82), ß _Playback “rain” call: phase after_ = −1.99 (−3.22, −0.82), Fig. 2a) and drastically increased the number of “chink” calls during the broadcast of both songs (Table 2, M2, ß _Playback song: phase after_ = 3.28 (0.42, 6.11)) and “rain” calls (Table 2, M2, ß _Playback “rain” call: phase after_ = 1.95 (−0.94, 4.82)). While the song rate resumed to pre-playback values after song broadcasts, it remained low after “rain” call broadcasts (Post-hoc test estimate: −0.96, 95% credible interval: −1.51, −0.41, Fig. 2b) although not statistically lower than before the playback. The number of “chink” calls during “rain” call playbacks was intermediate between that of control and song playbacks and remained consistent during and after the broadcast (Fig. 2b). We did not find strong statistical support that “rain” call playbacks elicited “rain” calls in chaffinches (Table 2, M3, ß _Playback “rain” call_ = 1.67 (−0.64, 4.14), ß _Playback song_ = −0.74 (−3.47, 1.8), Fig. 2c), however, birds that were already “rain” calling before the playback kept “rain” calling with very high probability (Table 2, M3, ß _“rain” call pre playback_ = 3 (0.11, 6.39)). Approach probability, latency, and minimum distance to the loudspeaker are typically considered as measures of aggressiveness in passerines. In all three parameters, the “rain” call playback elicited response intensities intermediate between song and control playbacks. The approach probability over time increased with only half the rate for “rain” calls (Table 2, M4, ß _Playback “rain” call_ = 1.93 (0.61, 3.46)) than for songs (Table 2, M4, ß _Playback song_ = 4.26 (2.97, 5.85), Fig. 2d), and the effect size of “rain” call playback on approached distance was half that of song playback (Table 2, M5, ß _Playback “rain” call_ = −4.59 (−7.52, −1.7), ß _Playback song_ = −8.82 (−11.5, −6.15), Fig. 2e). Whether or not the female was visible did not influence any of the chaffinches’ reaction to the playback. Birds that were further away at the start of the playback also stayed further away from the loudspeaker (Table 2, M3, ß _Initial distance_ = 0.65 (0.33, 0.96)).

**Table 2.** **Behavioral response of male chaffinches to the playback of “rain” calls and conspecific songs.** Model estimates (posterior mean) and 95% credible intervals (2.5 and 97.5 posterior distribution percentiles). Credible intervals not overlapping zero are highlighted in bold. Model numbers refer to Table 1.

	M1	M2	M3	M4	M5
Model	Number of complete songs	Presence of “rain” calls after playback start	Number of “chink” calls	Approach probability/latency	Minimum distance to loudspeaker
***Fixed effects***	***Estimate (95 % CrI)***				
**Intercept**	**1.14 (0.67, 1.59)**	−**2.96 (**−**5.48,** −**0.85)**	−**2.99 (**−**4.51,** −**1.61)**	−**6.62 (**−**8.68,** −**4.81)**	**14.01 (11.79, 16.17)**
**Rain” call playback**	0.09 (−0.41, 0.57)	1.67 (−0.64, 4.14)	**1.61 (0.03, 3.25)**	**1.93 (0.61, 3.46)**	−**4.59 (**−**7.52,** −**1.7)**
**Song playback**	0.42 (−0.05, 0.91)	−0.74 (−3.47, 1.8)	1.46 (−0.14, 3.11)	**4.26 (2.97, 5.85)**	−**8.82 (**−**11.5,** −**6.15)**
**During playback**	0.08 (−0.58, 0.76)		0.52 (−1.94, 2.99)		
**During “rain” call playback**	−**1.99 (**−**3.22,** −**0.82)**		1.95 (−0.94, 4.82)		
**During Song playback**	−**1.94 (**−**3.13,** −**0.82)**		**3.28 (0.42, 6.11)**		
**After Playback**	−0.27 (−0.76, 0.25)		−0.21 (−2.1, 1.72)		
**After “rain” call playback**	−0.56 (−1.29, 0.13)		1.76 (−0.49, 3.93)		
**After Song playback**	0.07 (−0.63, 0.76)		**3.53 (1.22, 5.92)**		
**Female present**	−**0.7 (**−**1.07,** −**0.34)**	0.11 (−2.99, 3.04)	0.46 (−0.41, 1.31)	0.07 (−1.07, 1.22)	−0.44 (−3.59, 2.53)
**“Rain” call during the pre-playback phase**		**3.0 (0.11, 6.39)**			
**Initial distance**	0.17 (−0.06, 0.41)	0.23 (−0.06, 0.55)	−0.33 (−0.96, 0.27)	0.04 (−0.1, 0.18)	**0.65 (0.33, 0.96)**
**Playback amplitude**	0.25 (−0.24, 0.71)	0.92 (−0.17, 2.07)	−1.04 (−2.39, 0.22)	−0.47 (−0.99, 0.02)	0.24 (−1.01, 1.44)
**Time to playback**	0.12 (−0.07, 0.33)	−**1.46 (**−**3.2,** −**0.04)**	−0.13 (−0.65, 0.38)	−**0.77 (**−**1.55,** −**0.04)**	**1.83 (0.49, 3.17)**
***Random effects***	***Standard deviation***				
**Bird ID**	0.51	0.43	0.71	0.43	0.55

**Fig. 3.**
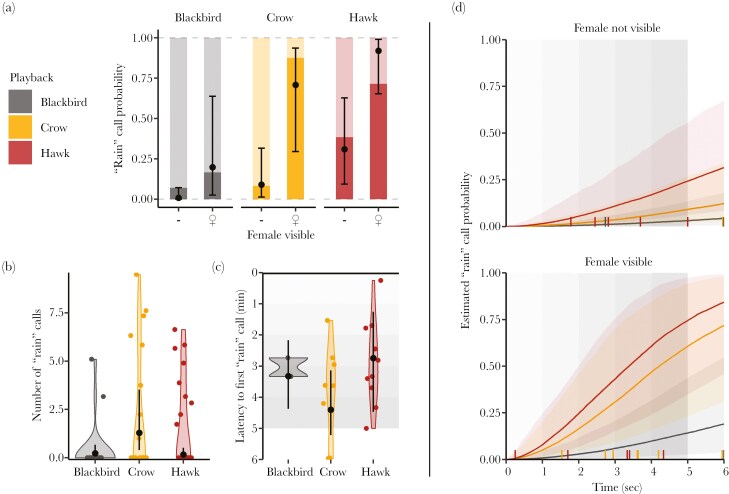
Chaffinches react to predator call playback with “rain” calls. a) Probability that birds used “rain” calls during the playback, in the presence or absence of a female detected by observers, b) number of “rain” calls (square-rooted), and c) latency to the first “rain” call for each playback type (color-coded). d) Predicted probability (posterior mean and 95% credible interval) that chaffinches use “rain” call over time in relation to the playback type (color-coded). a-c) Black dots and error bars depict the model mean posterior distribution and 95% credible interval. a) Raw proportion of the trials with “rain” calls (dark) or without “rain” calls (light) depicted by the bars in the background. b, c) Raw data (points) and data distribution (violin plots) are shown in the background. b, d) The playback period is grayed out, increasing danger is represented with a darker shade.

In Playback 2, we tested whether chaffinches started using “rain” calls in the presence of predators. “Rain” call probability increased drastically in the presence of crow calls (Table 3, M7, ß _Playback crow_ = 2.20 (0.13, 4.88)) and hawk calls (ß _Playback hawk_ = 3.80 (1.57, 6.57), Fig. 3a), compared to the control blackbird songs. Moreover, the presence of a female visible to observers during trial increased the “rain” call probability (Table 3, M7, ß _female_ = 3.25 (1.41, 5.65), Fig. 3a), and loud ambient noise tended to decrease it (Table 3, M7, ß _ambient noise_ = −1.17 (−2.35, −0.10)). Finally, the “rain” call probability tended to decrease when the birds flew more (typical feeding behavior, Table 3, M7, ß _flight activity_ = −0.62 (−1.75, 0.43)). Crow calls increased the normalized number of “rain” calls after playback (Table 3, M9, ß _playback crow_ = 1.71 (1.12, 2.35), Fig. 3b). However, both female detection by observers (ß _female visible_ = −0.84 (−1.40, −0.34)) and louder ambient noise (ß _Ambient noise_ = −2.35 (−2.90, −1.89)) decreased the normalized number of “rain” calls. The latency to the first “rain” call was higher with crow calls than for control songs (Table 3, M8, ß _Playback crow_ = 0.78 (0.28, 1.34), Fig. 3c), while hawk calls tended to decrease latencies (Table 3, M8, ß _Playback hawk_ = −0.40 (−1.30, 0.62), Fig. 3c). We also found that greater initial distances between the playback source and the focal bird led to longer latencies for “rain” calls (Table 3, M8, ß _Initial distance_ = 0.49 (0.34, 0.67)), while we failed to find a statistical effect of female detection by observers. There was a very strong inter-individual difference in “rain” call latency and number (Table 3, M9, Var _Bird ID_ = 1.65; M10, Var _Bird ID_ = 2.73). In the Time-to-Event model, “rain” call probability increased over time in the presence of hawk calls (M10, ß _Playback hawk_ = 2.22 (0.94, 3.75), Fig. 3d), but we found no statistical effect of crow calls on “rain” call probability over time. When we saw a female, the probability of “rain” call increased (Table 3, M10, ß _Female visible_ = 1.57 (0.59, 2.69), Fig. 3d, lower panel). “rain” call probability decreased with a larger distance to the speaker, albeit the effect size is about eight times lower than that of the playback type.

**Table 3.** **Behavioral response of male chaffinches to the playback of predator vocalizations.** Model estimates (posterior mean) and 95% credible intervals (2.5 and 97.5 posterior distribution percentiles). Credible intervals not overlapping with zero are highlighted in bold. Model numbers refer to Table 1.

	M6	M7	M8	M9
Model	Presence of “rain” calls	Latency to “rain” calls	Number of “rain” calls after playback start	Time to “rain” call
***Fixed effects***	***Estimate (95 % CrI)***			
**Intercept**	−**4.72 (**−**7.42,** −**2.56)**	**0.07 (**−**0.72, 0.82)**	−**3.03 (**−**4.31,** −**1.82)**	−**13.76 (**−**18.37,** −**9.78)**
**Crow playback**	**2.34 (0.26, 4.71)**	**0.79 (0.31, 1.30)**	**1.71 (1.09, 2.32)**	1.19 (−0.18, 2.74)
**Hawk playback**	**3.91 (1.66, 6.47)**	−0.39 (−1.20, 0.61)	−0.32 (−0.86, 0.20)	**2.22 (0.94, 3.75)**
**Female visible**	**3.30 (1.44, 5.58)**	0.02 (−1.10, 1.00)	−**0.85 (**−**1.38,** −**0.33)**	**1.57 (0.59, 2.69)**
**Flight activity**	−0.61 (−1.72, 0.39)			
**Time to first “rain” call**			**0.09 (0.07, 0.10)**	
**Initial distance**	−0.24, (−0.53, 0.0)	**0.50 (0.34, 0.69)**	**0.48 (0.37, 0.60)**	−**0.14 (**−**0.30,** −**0.01)**
**Ambient noise**	−**1.17 (**−**2.33,** −**0.13)**	−**1.98 (**−**2.59,** −**1.44)**	−**2.38 (**−**2.91,** −**1.90)**	−0.41 (−1.07, 0.23)
***Random effects***	***Standard deviation***			
**Bird ID**	0.22	1.29	1.66	0.11

**Fig. 4.**
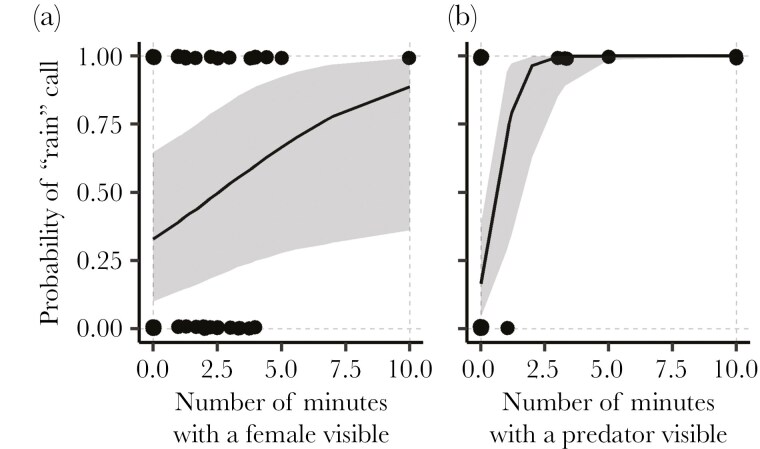
Chaffinches use “rain” calls more often in the presence of a female or a predator. Predicted probability (posterior mean and 95% credible interval) of “rain” calls production during the 10-min behavioral observation blocks as a function of the number of minutes with (a) females present or (b) predators present. Black dots represent the raw data.

Finally, we observed five males to determine the context in which “rain” calls occur spontaneously. The males were likely paired, as nest-building activity and mate-guarding were observed in all territories and copulations in four out of five. Conspecific male presence (song or physical presence) did not affect “rain” call probability. In contrast, whether we saw a female increased the probability of hearing “rain” calls (ß _Female visible_ = 2.82 (0.21, 5.73), Fig. 4a). Similarly, when predators were detected, “rain” calls were far more likely (by about one order of magnitude, ß _Predator_ = 24.66 (12.16, 40.92), Fig. 4b).

## Discussion

We combined playback experiments with behavioral observations to investigate the function of the “rain” call of the chaffinch. In particular, we tested three mutually non-exclusive hypotheses, according to which the “rain” call could be (1) a substitute for song in territorial defense, (2) an alarm call, or (3) a within-pair communication call. We found that male chaffinches responded differently to the playback of “rain” calls than to song playback. In addition, chaffinches started uttering “rain” calls earlier and considerably more often after the playback of predator calls or when actual predators were present, which supports the alarm-call hypothesis. Third, chaffinches were more likely to emit “rain” calls when a female was visible nearby, which is consistent with the within-pair communication hypothesis.

The most striking difference in the behavior of the tested males in response to “rain” calls compared to songs was that songs immediately elicited a strong aggressive response (high “chink” call rate, very fast approach to a close distance), while “rain” calls triggered much weaker responses and the tested chaffinches did not resume singing activity after “rain” call playbacks. This finding does not support the idea that “rain” calls are fully equivalent to songs. Passerines sometimes use different signals during a graded territorial response (Hof and Podos 2013), and the much lower response of birds to “rain” calls than to songs might indicate that the “rain” calls might be used in less aggressive contexts. However the “chink” call is typical of both aggressive interactions—such as territorial disputes (Marler 1956b; Budka et al. 2019)—and predator alarm or mobbing (Sick 1939; Marler 1956b; Randler and Förschler 2011). Therefore, the response to “rain” calls could also be interpreted differently, as the birds may have responded to “rain” calls as they would respond to the presence of danger. Contrarily to song playbacks, the birds did not resume singing after “rain” call playbacks, which could indicate the perceived presence of a predator. Further strong support for the alarm-call hypothesis comes from both our behavioral observations and the predator call playback: not only did we detect a correlation between the occurrence of “rain” calls and the presence of actual wild predators, we were also able to consistently trigger “rain” call production by presenting predator cues. While the correlation between the occurrence of “rain” calls and the presence of predators could potentially be affected by an observer-expectancy bias if observers look more thoroughly for predators when they hear “rain” calls, the experimental evidence obtained with the second playback was not affected by such a potential caveat.

Interestingly, the audience affected the chaffinches’ decision and latency to “rain” call in response to a predator, as males were more likely to “rain” call when their mate was visible nearby. If the female was not visible to observers, this does not necessarily mean that the male didn’t know where she was. Accordingly, this uncertainty may add noise to the data. On the other hand, the effect of female presence eliciting “rain” calling was observed not only in our predator playback but also in a previous behavioral observation (de Framond et al. 2024) as well as an anecdotal report (Marler 1956b), indicating that the audience effect on “rain” call production is not simply a product of sampling bias. Further investigation is necessary to ascertain the exact role of females in the males’ motivation to produce “rain” calls. Audience effects are known to modulate alarm-calling behavior in many group-living animals, sometimes giving rise to specialized sentinel systems (Blumstein et al. 1997; Wheeler 2008; Gill and Bierema 2013; Bednekoff 2015; McLachlan et al. 2019). For example, domestic fowl (*Gallus gallus*) produce alarm calls almost exclusively when conspecifics are visible (Karakashian et al. 1988). The same effect has been reported for red-legged partridge (*Alectoris rufa*) males, which, in addition, increase their alarm call rate when a female is around compared to an unfamiliar male (Zaccaroni et al. 2013). It has been suggested that alarm-calling behavior is regulated by a trade-off between potential fitness benefits and increased risk of being detected by the predator (Taylor et al. 1990). “Rain” calls only occur during the breeding season, more often when females were visible, and in the presence of predators that are not dangerous for adult chaffinches (ie carrion crows), hence we can hypothesize that “rain” calls are part of a defense strategy to optimize fitness, for example against nest/chick predation. This would explain why nestlings fall silent when they hear “rain” calls (Marler 1956a; Davies et al. 2004), and some of the differences observed in the “rain” call activity in response to crows and hawks. The “rain” call probability and the latency to the first “rain” call scaled with the risk posed by predator identity: “rain” calls were more frequent and uttered earlier in response to hawks, which are dangerous for both adults and nestlings (Newton 2010), than in response to crows, which are dangerous for nests only. In contrast, the number of “rain” calls was highest for the predator most likely to attack the nest, ie the crow.

In the same experiment, “rain” call latency scaled strongly with ambient noise: an increasing noise level led to fewer “rain” calls, but the males also started calling earlier. The latter is surprising because noise has been found to delay the production of alarm calls in birds (Courter et al. 2020) and also impair their perception (Templeton et al. 2016). Generally, the noise levels at our study sites were not especially high and mainly due to other birds singing, including other chaffinches. The shorter reaction time of birds exposed to higher background noise could be explained by many factors, including increased vigilance (Quinn et al. 2006), the “flock of two” effect (Lima 2009), or even the spatial distribution of different personality types. Risk-taking in predation defense is known to be part of a suite of behaviors that consistently co-vary (ie personality, Hollander et al. 2008), and we detected very strong inter-territory differences in the latency to “rain” call. As we did not work with a marked population, we cannot ascertain that we measured repeatedly the same individuals. However, chaffinches are known to be very territorial and with stable territories over the season, therefore, sampling a bird at the same location within a few days is very likely to yield repeated measures.

Since only male chaffinches emit “rain” calls, they seem to bear most of the responsibility for predator surveillance, while females are responsible for nest building, incubation, and nestling rearing (Clement et al. 2023). Sex-specific division of labor is one of the most well-studied consequences of sexual asymmetry with males usually involved in territorial defense and females responsible for parental care (Andersson 1994; Queller 1997; Webb et al. 2010). While both parents typically join nest predator defense or mobbing (Teunissen et al. 2020), the “flock of two” hypothesis postulates that two individuals of a breeding pair behave as a flock, with one animal being a sentinel while the other gains time to feed, preen or give parental care (Lima 2009). A notable example is the alarm system of breeding red-winged blackbirds (*Agelaius phoeniceus*), in which males emit alarm calls almost continuously, changing both call type and rate to indicate the presence and urgency of danger (Beletsky 1991). In the yellow warbler (*Dendroica petechia*), males have distinct calls that refer specifically to brood parasites and prompt the female to rush to the nest (Gill and Sealy 2003). In chaffinches, the “rain” call is an alarm call that is produced by males during the breeding season; therefore, we propose that the “rain” call is functionally equivalent to the distinct nest-danger alarm system of red-winged blackbirds and yellow warblers.

Chaffinch “rain” calls have strongly differentiated dialects, their implication for communication remains to be determined. We were only able to match the “rain” call dialect of the playback to that of focal birds in four cases, and the low sample size did not allow us to perform statistical analyses. We did not observe that these four birds reacted differently than the other birds (see Fig. S2), but we cannot exclude a potential effect of the dialect on the reaction strength. Several birds in our study used the two dialects either alternatively across the breeding season or concomitantly within the same bout, and one bird even switched dialect from the “huit” type before playback to “rülsch” call, ie the playback dialect, during the trials. This indicates that a male chaffinches can understand both dialects. Anyhow, our results of both the behavioral observations and the predator call playback were not affected by any mismatch of the dialect type.

Collectively, our findings suggest that the “rain” call is likely an alarm call. This begs for an interesting evolutionary question, given the unusual characteristics of this call with learned dialects and a seasonal production (potentially based on the same hormonal control as song). What are the proximate and ultimate causes underling the evolution of an alarm call with learned components? Vocal learning enables birds to acquire particularly large and varied song repertoires, which, at least in some species, may be under sexual selection (Catchpole and Slater 2008). In addition, in species where nest predation is exceptionally high, such as the chaffinch (Hanski and Laurila 1993), having more differentiated alarm calls (ie alarm calls for general predators and for nest predators) might have fitness benefits. There are at least two other examples of alarm call system that are likely under sexual selection: In fowl, efficient alarm calling behavior was found to be a good predictor for reproductive success, and it functions in advertising male quality both directly and indirectly (Wilson et al. 2008). In males of social mammals, such as the guenons, the originally naturally selected alarm system against predators was shaped by sexual selection to become also important for intra-sexual competition and mate choice (Zuberbühler 2002). While these examples are only remotely connected to the chaffinch case, further investigation of the fitness consequences and female responses to “rain” calls in general and different “rain” call dialects in particular would be instrumental to understand the communication system of this very common species.

In conclusion, we tested three hypotheses to explain the function of the chaffinch “rain” call. In line with the notion of a nest-predator alarm call, the “rain” call is consistently elicited by predator presence, and emitted more often when females are in sight. While “rain” calls are no direct song substitute, they might be under sexual selection, which could explain their song-like features. A combination of high nest predation pressure and sexual selection might drive the emergence of complex alarm call systems, and investigating the role of females in these systems would be crucial to understand their ecology and evolution.

## Supplementary Material

araf039_suppl_Supplementary_Materials_1

## Data Availability

Analyses reported in this article can be reproduced using the data provided by de Framond et al. (2025).
