# Supplementary material for: Decoding the chaffinch “rain” call: a female-directed alarm call?
Source: Behav Ecol. 2025 May 4;36(4):araf039. doi: 10.1093/beheco/araf039 (PMC12137894; doi:10.1093/beheco/araf039)
Supplement: araf039_suppl_Supplementary_Materials_1 [file araf039_suppl_supplementary_materials_1.docx]

**Supplements for:**

**Decoding the chaffinch "rain" call: a female-directed alarm call?**

**Contents:**

Figure S1: Spectrograms of the different playback types

Figure S2: Chaffinches with “rain” call dialect matching and not matching the playback did not respond differently.

**
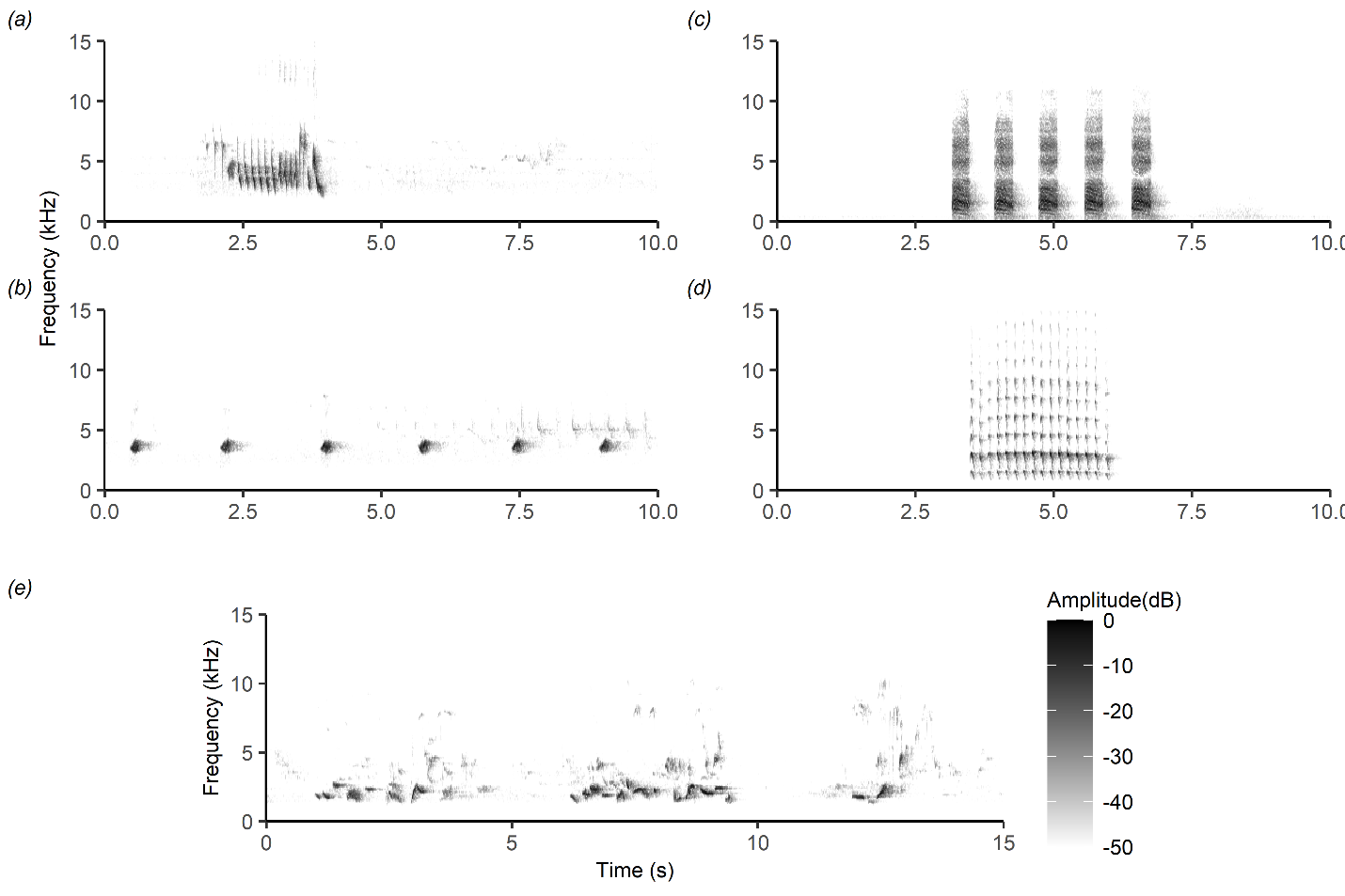
**

**Figure S1: Spectrogram of the different playback types.** a) Chaffinch song, b) chaffinch rain calls, c) crow calls (*Corvus corone*), d) sparrowhawk calls (*Accipiter gentilis*), e) blackbird song (*Turdus merula*). Only a short portion (10-15 seconds) of each playback type is shown as an example.

**
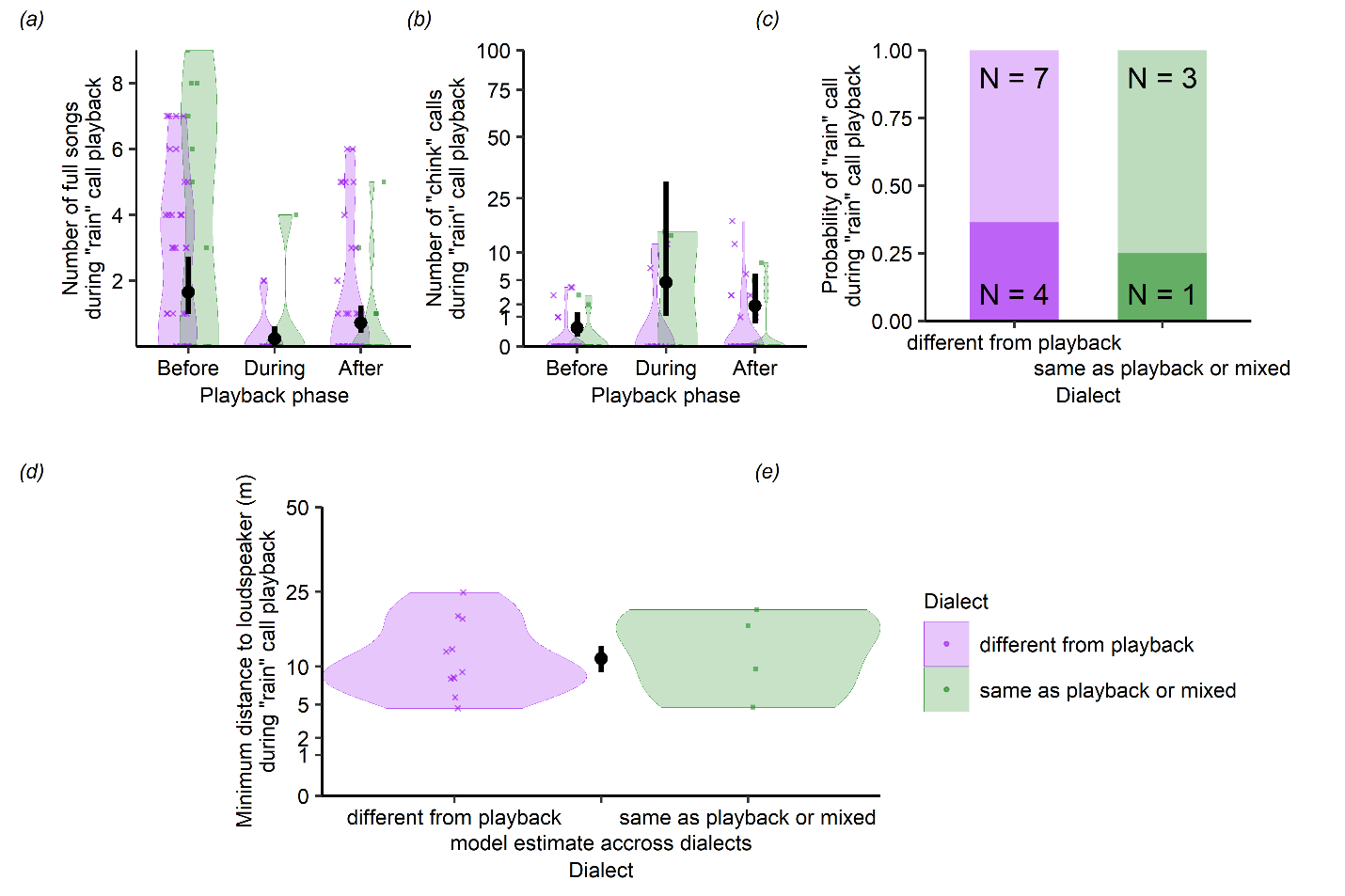
**

**Figure S2: Chaffinches with “rain” call dialect matching (green) and not matching (lilac) the playback did not respond differently.**

Because of the low sample size we could not add the playback type into the models. This figure is the pendant of figure 2 in the manuscript, splitting the response of birds to “rain” call playback according to whether the dialect of the focal birds was the same than that of the playback or not. a) Number of full songs and b) of “chink” calls during “rain” call playbacks according to playback phase. C) proportion of birds that used “rain” calls during or after the presentation of “rain” calls (sample size in the bars, dark bars represent birds that did use “rain” calls). d) Minimum distance to the loudspeaker according to dialect type. We added in black the estimate and 95% credible interval from the models run across dialect types, for comparison.
